# Reviewing the Landscape of Cancer Survivorship: Insights from Dr. Lidia Schapira’s Programs and Beyond

**DOI:** 10.3390/cancers16061216

**Published:** 2024-03-20

**Authors:** Viviana Cortiana, Rabab Hunaid Abbas, Soumiya Nadar, Diksha Mahendru, Jade Gambill, Gayathri Pramil Menon, Chandler H. Park, Yan Leyfman

**Affiliations:** 1 Department of Medical and Surgical Sciences (DIMEC), University of Bologna, 40126 Bologna, Italy; 2 Tbilisi State Medical University, 0186 Tbilisi, Georgia; rabababbas2002@gmail.com (R.H.A.);; 3 Global Remote Research Scholars Program, St. Paul, MN 55101, USA; 4 Parker University, Dallas, TX 75229, USA; jade.l.gambill@outlook.com; 5 Norton Cancer Institute, Louisville, KY 40202, USA; chandler.park@louisville.edu; 6 Icahn School of Medicine at Mount Sinai, New York, NY 10025, USA; yan.leyfman@mssm.edu

**Keywords:** cancer survivorship, cancer care, oncology, cancer patient journey, precision medicine, mental health and cancer

## Abstract

**Simple Summary:**

This review begins by exploring the escalating global population of cancer survivors, drawing inspiration from Dr. Lidia Schapira’s Keynote Conference on Survivorship 1.0 and Survivorship 2.0 Programs. It presents and discusses the transformed and constantly evolving landscape of cancer care, emphasizing patient-centric strategies within Cancer Survivorship Programs, including connection, support, and education. While spotlighting cancer recurrence surveillance, concerns arise regarding potential oversights in addressing the enduring mental and physical health impacts. The study further navigates mental health challenges faced by survivors providing strategies to mitigate them, insights into promising research areas, such as Precision Medicine’s role in de-escalating oncology therapies, as well as advocating for early cancer awareness and referrals to supportive services. Dr. Schapira’s insights also extend to examining online resources, emphasizing their role in educating healthcare practitioners and future generations in cancer care. Additionally, the paper aims to identify knowledge gaps in cancer care and envision future developments toward accurate, holistic care, improving survivor quality of life, and enhancing mental and physical well-being.

**Abstract:**

The constantly escalating population of cancer survivors worldwide has prompted a focused exploration of their unique needs and experiences within the context of healthcare medicine. This review initiates its analysis inspired by Dr. Lidia Schapira’s insightful keynote conference on the Survivorship 1.0 and Survivorship 2.0 Programs, shedding light on their implementation challenges and setting the stage for a comprehensive analysis of cancer survivorship initiatives. Within the transformed landscape of cancer care, patient-centric strategies embedded in cancer survivorship programs comprising vital elements such as connection, support, and education are presented. While placing cancer recurrence surveillance at the forefront, the review underlines concern regarding the potential oversight of the enduring impact on mental and physical health. Dr. Schapira’s insights further extend into the exploration of mental health challenges faced by survivors, promoting an examination of diverse strategies to address these concerns. Furthermore, the discussion continues toward promising areas of research, notably Precision Medicine’s role in de-escalating cancer therapies, and advocates for measures such as early cancer awareness and timely referrals to supportive services. Highlighting the significance of education, the role of online resources in enhancing the knowledge of healthcare practitioners and future generations in cancer care is then explored. The paper concludes by presenting some of the most prominent global current survivorship programs, identifying critical knowledge gaps in cancer care and projecting future developments aimed at delivering accurate and holistic care, improving the quality of life for survivors, and enhancing both mental and physical well-being. Drawing upon the insights from Dr. Schapira, this review lays the groundwork for a nuanced exploration of cancer survivorship and its multifaceted implications.

## 1. Introduction

In the context of healthcare, the concept of “Cancer Survivorship” transcends its initial characterization as a mere medical term, evolving into a comprehensive framework that encapsulates the intricate journey beginning with a diagnosis and potentially extending throughout a lifetime [1]. Rooted in an unwavering commitment to addressing the holistic well-being of cancer survivors, this dynamic field places a paramount focus on understanding and meeting the unique needs and experiences of individuals navigating life with, during, and beyond cancer.

Historically, a cancer diagnosis has frequently been associated with a grim prognosis, focusing primarily on diagnostic procedures, treatment interventions, and palliative care for most advanced cases. However, the landscape of cancer care has undergone a remarkable transformation, leading to a paradigm shift with a more optimistic outlook for many patients. The term “survivorship” has, therefore, gained prominence as a result of significant strides in oncology research and treatment, contributing to noteworthy improvements in survival rates across various types of cancer. The progress is underscored by compelling numerical data indicating enhanced outcomes for patients. For instance, recent studies have revealed a substantial increase in the overall five-year survival rate for cancer patients over the past decade, reaching 69%. Over the next decade, the number of people who have lived 5 or more years after their cancer diagnosis is projected to further increase by approximately 30%, to 16.3 million [2]. Breakthroughs in targeted therapies and immunotherapies have further propelled advancements in survival rates for specific cancer types. Notably, localized breast cancer now boasts an estimated 99% five-year survival rate [3]. The once-challenging landscape of lung cancer has also witnessed notable progress, particularly with the introduction of targeted therapies and immunotherapies, resulting in improved outcomes and an increased five-year survival rate for certain subtypes [4]. Similarly, melanoma, a type of skin cancer, has seen remarkable improvements in survival rates, reaching more than 99% for localized cases [5,6]. These numerical advancements reflect not only the efficacy of contemporary treatment modalities but also an evolving landscape, with a growing number of individuals successfully completing their treatment and transitioning into the phase of survivorship. The evolving nature of survival rates underscores the importance of ongoing research and innovative treatment approaches in further optimizing outcomes for individuals affected by cancer.

The widespread adoption of the term “survivor” in the context of cancer is attributed to Dr. Fitzhugh Mullan, a pivotal figure whose influential essay, “Seasons of Survival: Reflections of a Physician with Cancer”, was published in 1985 [7]. His groundbreaking work challenged the prevailing notion that the cancer experience was confined to the acute phases of diagnosis and treatment. Instead, he advocated how the impact of cancer transcends these initial stages, shaping the emotional and psychological landscape of individuals well beyond the immediate challenges of medical intervention. In recounting his own personal battle with cancer, Dr. Mullan highlighted the intricate emotional and psychological stages, shedding light on the profound and enduring effects of the cancer journey on an individual’s life. His narrative not only provided a comprehensive understanding of the multifaceted dimensions of survivorship but also played a crucial role in reshaping societal perceptions and promoting a more empathetic and holistic approach towards those affected by cancer. The legacy of his work as a light, guiding individuals, healthcare professionals, and the broader community in recognizing and addressing the complexities of the cancer experience.

In the dynamic landscape of cancer care shaped by continuous advancements in medical science, a significant paradigm shift has unfolded, challenging the traditional understanding of survivorship. Beyond the conventional focus on the trajectory through treatment, the contemporary perspective recognizes that it extends into the intricate tapestry of daily life, marking a profound evolution in the holistic approach to cancer survivors. A pivotal aspect of this transformative paradigm is the emergence of the “Silver Tsunami” phenomenon [8], a term that encapsulates a demographic shift within the cancer survivor population. This change is characterized by a predominant representation of older individuals who have successfully overcome the challenges of cancer. This occurrence not only symbolizes the increasing number of older survivors but also underscores the unique challenges they bring to the survivorship landscape. Because this demographic brings not only a history of cancer but also the complexities of comorbidities associated with the aging process, the healthcare system is compelled to adapt and tailor survivorship care to address the specific needs of this growing population. This shift emphasizes the need for comprehensive and multidisciplinary approaches that go beyond the traditional boundaries of cancer treatment, considering the multifaceted aspects of aging and survivorship. The “Silver Tsunami” phenomenon is a critical lens through which researchers, healthcare providers, and policymakers must view the evolving landscape of cancer survivorship, thereby facilitating a deeper understanding and more tailored interventions for this unique and growing demographic.

**Figure 1:**
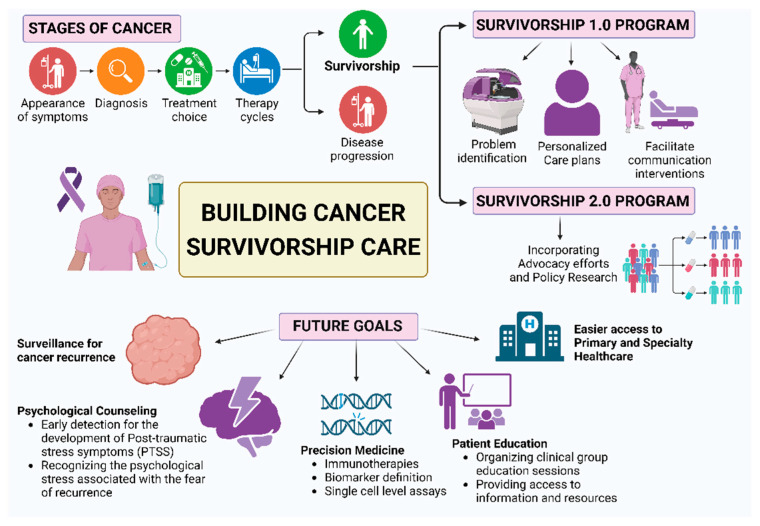
The key elements necessary for building a cancer survivorship program are highlighted, and the 2 main programs implemented in the healthcare field along, with their virtues, are presented. Created with BioRender.com.

As we embark on a deeper exploration of cancer survivorship, the evolving understanding emphasizes the importance of navigating the seas of survivorship with compassion, precision, and a profound acknowledgment of the lives reshaped by the journey through and beyond cancer. This paper aims to explore the multifaceted concept of cancer survivorship within the healthcare setting, tracing its historical progression from a grim prognosis to the current paradigm shift that recognizes it extending beyond treatment. The goal is to highlight the profound journey individuals undertake from the moment of diagnosis, through the challenges of therapy, and into the intricate post-treatment phase (Figure 1). 

## 2. Evolution in Cancer Survivorship Care: From Survivorship 1.0 to Survivorship 2.0

With the global implementation of diverse programs, the provision of care for cancer survivors has undergone significant transformation. This evolution is a multifaceted journey that has seen significant advancements, particularly marked by the distinctive phases also denoted as Survivorship 1.0 and Survivorship 2.0.

Survivorship 1.0, a seminal period in oncology that began around 2005, represented a paradigm shift from traditional cancer care approaches. This phase originated from the influential book “From Cancer Patients to Cancer Survivors” [9], which was inspired by pediatric models, and primarily centered around dedicated clinics committed to addressing immediate post-treatment concerns. During this transformative phase, Survivorship 1.0 emphasized the need for a comprehensive approach to this specific care, shedding light on key concerns such as the absence of standardized therapy plans, insufficient communication among healthcare providers, and the need for better coordination of care [10]. The term “lost in transition” aptly encapsulates the challenges faced by many survivors, experiencing a complex healthcare landscape without clear guidance on managing the long-term effects of cancer and its treatment.

Survivorship 1.0 saw a notable emphasis on post-treatment care, introducing tailored plans and continued medical follow-up to monitor the potential late effects of treatments and address the risk of recurrence. This phase extended beyond medical considerations, leading to a broader understanding of survivorship and incorporating psychosocial and emotional well-being as essential components. Quality of life became a central concern during Survivorship 1.0, prompting the development of strategies to manage the multifaceted challenges faced by patients.

The term “Cancer Survivorship 1.0” encapsulates the juncture at which the medical community and society recognized and responded to the evolving needs of those who had completed their primary cancer treatments. This acknowledgment then set the stage for further advancements in the ongoing dialogue surrounding cancer survivorship. Until 2018, dedicated programs predominantly focused on clinics, addressing immediate concerns but exhibiting limitations in comprehensive inclusion.

From these premises, recognizing the need for a more inclusive and comprehensive approach, Survivorship 2.0 emerged as a pivotal concept in the ongoing evolution of cancer survivorship care. Highlighted by Eva Grunfeld [11], Survivorship 2.0 emphasizes the multifaceted dimensions of this phase for patients and urges the implementation of programs that can address diverse needs more effectively. This initiative, therefore, marked a significant shift, integrating clinical aspects with advocacy efforts and policy research, challenging healthcare systems to adapt and evolve [12].

However, the implementation of Survivorship 2.0 was not without challenges. The transition from the established Survivorship 1.0 model required significant changes in healthcare systems and practices. Potential drawbacks, including increased resource demands and the need for extensive training and education, raised several concerns among healthcare professionals and administrators.

To objectively assess the impact of Survivorship 2.0, an online cancer survivorship course designed to promote practice change among primary care clinicians conducted a detailed evaluation. The course, as outlined by Alberto et al. in their study on educational innovation, successfully employed asynchronous formats, employing patient-centered stories to effectively address knowledge gaps in cancer survivorship care [13]. The findings indicate that learners not only reported positive self-initiated changes in their practices but also revealed interesting insights about the integration of cancer survivorship principles into primary care.

In conclusion, this evaluation of Survivorship 2.0 underscores its effectiveness in educating primary care clinicians and promoting positive changes in their practices concerning cancer survivorship care. The incorporation of patient-centered stories and the reported self-initiated practice changes by learners highlight the course’s impactful educational innovation. However, recognizing the need for a more in-depth analysis, as emphasized by the authors, is crucial to fully comprehend the sustained and long-term implications on clinical practices. This study, therefore, significantly contributes to the evolving landscape of cancer survivorship education in primary care, emphasizing the ongoing importance of evaluating and improving such educational initiatives for optimal impact.

## 3. Revolutionizing Cancer Survivorship: A Holistic Approach to Care Integration

In recent years, the landscape of cancer care has undergone a profound transformation, marked by significant advancements in both prevention and treatment, resulting in a notable decline in mortality rates [14]. This transformative shift necessitates a reevaluation and enhancement of survivorship care, particularly for individuals who have successfully overcome the challenges of cancer. A pivotal component of this evolved approach involves actively engaging patients in their healthcare journey, emphasizing preventive measures and regular screenings to ensure early detection and intervention [15]. The cornerstone of survivorship care therefore lies in meticulous surveillance for cancer recurrence, extending beyond routine monitoring to consider each patient’s unique medical history and treatment response. This personalized approach not only enhances clinical effectiveness but also contributes to fostering a compassionate and supportive environment, fundamental for patients.

Therefore, a thorough risk assessment that guides tailored interventions, taking into account individual needs, is integral to this comprehensive strategy. This multifaceted approach ensures that survivorship care goes beyond clinical aspects, incorporating a strong focus on promoting positive behavioral changes. This includes detailed evaluations of smoking history and the encouragement of holistic lifestyle choices, such as incorporating regular exercise into daily routines. These behavioral interventions are aimed not only at enhancing the physical well-being of survivors but also at cultivating a sustainable and resilient post-cancer life. The integration of risk-stratified approaches within the survivorship program further amplifies its efficacy, offering precise and personalized care through genetic evaluation and targeted screening for second primary cancers based on individual exposure profiles. This forward-looking approach ensures that interventions are precisely aligned with each patient’s specific risks and needs, optimizing the program’s overall impact [16].

Addressing the long-term effects of cancer therapies appears paramount in survivorship care. Conditions experienced by patients, such as chemotherapy-induced peripheral neuropathy require ongoing assessment due to their persistent nature. Comprehensive monitoring and education on late effects, which may manifest even years post-treatment, are crucial components of this specialized care, equipping survivors with the knowledge and resources needed to navigate potential challenges in the long term.

Also recognizing the psychosocial dimensions of survivorship, particularly the psychological stress associated with the fear of recurrence, is a pivotal element of this comprehensive strategy. A study highlighting this aspect was conducted, with the specific aim to assess post-traumatic stress disorder in young breast cancer survivors [17]. This investigation was a prospective cohort research that enrolled 1302 women diagnosed with breast cancer at age < 40. Participants completed serial surveys, and additional information was gathered from a medical record review. At the study baseline (median, 5 months post diagnosis), socio-demographics, anxiety and depression, social support, mental comorbidities, and medications were all evaluated. The prevalence of PTSS was 6.3% out of 700 women with stage 1–3 illness. PTSS was inversely related to having a college degree and stronger social support. Posttraumatic stress symptoms (PTSS) are associated with significant morbidity and mortality, and identifying factors related to PTSS can help healthcare practitioners identify individuals at increased risk of developing psychopathology as a late sequela of cancer. The early detection of those at risk may facilitate personalized screening measures for the development of PTSS, as well as specific medical therapies to improve the mental health and quality of life of breast cancer survivors who were diagnosed at a young age.

This aspect is approached through a patient-centric lens within the cancer survivorship program, emphasizing collaboration between specialists and primary care clinicians. This collaboration ensures that survivors receive not only expert medical care but also the necessary support to address the holistic aspects of their well-being.

This detailed and comprehensive survivorship care strategy therefore comprises a multifaceted approach, addressing clinical, behavioral, genetic, and psychosocial elements [18]. By tailoring interventions to individual needs and fostering a collaborative and patient-centric environment, survivorship care becomes a dynamic and impactful continuum in the overall landscape of cancer care. As we dig deeper into the multifaceted dimensions of this comprehensive care strategy, it becomes evident that its essence lies in recognizing the dynamic and evolving needs of individuals post-cancer treatment.

In a crucial study investigating coping methods and anxiety among young breast cancer survivors [19], it was revealed that these individuals heavily rely on the support of family and friends. The research, which examined 833 women diagnosed with stage 0–3 breast cancer, reported a median age at diagnosis of 37 years (range: 17–40). Notably, at both 6 and 18 months post-diagnosis, social supports emerged as the most frequently cited coping mechanisms. A significant majority expressed moderate to substantial reliance on emotional support from various sources, including partners (90%), parents (78%), other family members (79%), and friends (88%). These findings underscore the imperative consideration of patients’ social networks when formulating survivorship therapies.

The study, therefore, contributes valuable insights emphasizing the importance of recognizing and integrating social support structures into personalized care plans. The collaborative approach within the cancer survivorship program aligns with such findings, as it ensures that patients not only receive expert medical care but also the necessary support to address the holistic aspects of their well-being.

In conclusion, the engagement of patients as active participants in their healthcare journey is not merely a procedural step but a fundamental shift toward empowering survivors to take charge of their well-being. Preventive measures and regular screenings emerge as crucial in this strategy, underlining the importance of early detection and intervention. The traditional concept of surveillance for cancer recurrence is thus elevated to a personalized level, at which each patient’s unique medical history and treatment response are meticulously considered. This not only enhances the clinical efficacy of survivorship care but also contributes to creating a compassionate and supportive environment that acknowledges the individuality of each patient’s unique journey. The comprehensive risk assessment integrated into the survivorship care strategy represents a fundamental departure from a one-size-fits-all approach. Tailored interventions, considering individual needs, become the hallmark of this multifaceted strategy, which extends beyond the clinical setting and further into the behavioral aspects of survivorship.

## 4. Evolution of Cancer Survivorship Care: Navigating a Dynamic Landscape through the Literature, Patient Perspectives, and Technology Integration

The profound transformation that the landscape of cancer survivorship care is undergoing is a subject of current interest, explored through several reviews, active engagement in online discussions, and careful analysis of emerging trends.

Dr. Schapira’s systematic review stands as a foundational piece, shedding light on the limitations of conventional cancer survivorship care plans (SCPs) [20,21].

Expanding beyond PubMed and exploring diverse online resources reveals a discourse that surpasses traditional academic boundaries. Patient advocacy groups and healthcare organizations play a pivotal role in this ongoing dialogue, offering diverse perspectives on the evolving role of survivorship care. Amplifying patient voices through online forums and social media platforms adds a unique, humanizing dimension to the conversation. These narratives, shared by cancer survivors, thus become powerful testimonies highlighting experiences with SCPs and underscoring the need for a more patient-centered and personalized approach [22]. In-depth Google searches yield insights from patient-centric organizations, emphasizing the need to actively involve survivors in decision-making processes. The prevailing sentiment calls for a departure from standardized models, urging the adoption of a nuanced and adaptive survivorship care approach that considers individual needs, preferences, and challenges.

Simultaneously, amidst the emphasis on survivorship consultations, healthcare professionals explore innovative technologies to elevate care. Telehealth platforms and mobile applications aim to facilitate continuous, technologically augmented communication between survivors and healthcare providers, addressing geographical distances and accessibility issues. Recent conferences and symposiums dedicated to cancer survivorship provide a platform for stakeholders, including healthcare providers, researchers, policymakers, and patient advocates. These forums discuss comprehensive survivorship care models that extend beyond medical aspects, incorporating a holistic framework covering psychosocial, emotional, and lifestyle factors. Google trends highlight a growing interest in holistic survivorship care approaches, emphasizing mental health, nutrition, and physical well-being post-treatment. As the healthcare landscape evolves, there is a clear acknowledgment of the intricate interrelatedness of factors influencing the survivorship journey.

While SCPs may not show a statistically significant improvement in health outcomes, the renewed focus on survivorship consultations does not diminish the relevance of structured care plans. Instead, the discourse pivots towards a dynamic, adaptive model that emphasizes continual refinement and customization based on individual needs, preferences, and evolving circumstances.

The evolving narrative within the field suggests a departure from rigidity and standardization toward a more fluid, patient-centric, and personalized model of survivorship care. This comprehensive exploration paints a vivid, evolving, and comprehensive picture of the cancer survivorship care landscape, shaped by patient perspectives, technological advancements, and collaborative efforts within the healthcare community. The quest for optimal survivorship care transcends the boundaries of traditional care plans, embracing a holistic and patient-centered paradigm. In conclusion, the discourse surrounding cancer survivorship care is dynamic and intricate, encompassing influences from academic research and patient advocacy to technological innovation and interdisciplinary collaboration [23]. Each facet contributes to a rich tapestry that underscores the need for a more personalized, patient-centered, and comprehensive approach to survivorship care. Also, by navigating this complex landscape, it is evident that the pursuit of optimal survivorship care extends beyond traditional care plans, marking a strong shift towards a more adaptive, inclusive, and technologically integrated model resonating with the diverse and interconnected experiences of cancer survivors [24].

## 5. Towards Shaping the Future of Cancer Survivorship: Current Global Holistic Care Programs

Within the evolving landscape of cancer survivorship, the paradigm is already actively and constantly leading towards a holistic approach that extends beyond treatment phases. Acknowledging the unique needs of survivors, a multitude of programs has emerged globally, showcasing a commitment to comprehensive care playing fundamental roles in enhancing the post-treatment experience for cancer survivors. Programs from renowned institutions like Memorial Sloan Kettering Cancer Center to global foundations like Livestrong exemplify the diverse efforts aimed at providing personalized care, evidence-based guidelines, and a supportive community. Noteworthy contributions to survivorship care also come from organizations such as Cancer Care Ontario (CCO), which provides evidence-based guidelines to ensure continuity of care in Canada, the European Cancer Organization (ECCO), offering guidelines focused on the European context, and Cancer Australia, which develops tailored survivorship care guidelines for the Australian healthcare system. In the UK, the National Institute for Health and Care Excellence (NICE) provides comprehensive clinical guidelines covering various aspects of cancer care, including survivorship. These collective endeavors, which are supported by guidelines from multiple leading global organizations, shape the trajectory of survivorship care on a worldwide scale [25].

### 5.1. Livestrong Cancer Institute Survivorship Program

The Livestrong Cancer Institute Survivorship Program, initiated by the Livestrong Foundation, stands as a cornerstone in the global landscape of cancer survivorship support. Launched with a vision to provide a comprehensive network of services for individuals experiencing life post-cancer treatment, this program addresses a spectrum of challenges considering physical, emotional, and practical dimensions. Livestrong’s commitment to a holistic approach underlines the recognition that the post-treatment journey involves not only medical aspects but also aspects of emotional well-being and practical considerations. In line with their mission, the program offers an extensive range of resources tailored to meet the diverse needs of cancer survivors across the globe. These may include personalized counseling, rehabilitation services, and educational materials aimed at empowering patients with the knowledge and tools necessary to reclaim their lives. By fostering a global network of support, Livestrong ensures that survivors can connect with others who share similar experiences, creating a sense of community that plays a crucial role also in the healing process. Moreover, Livestrong’s Survivorship Program reflects a commitment to evidence-based practices, aligning with the broader trend in the evolving landscape of cancer care. By integrating the latest research findings and medical insights into their survivorship initiatives, Livestrong strives to provide cutting-edge and effective support strategies for individuals transitioning into life beyond cancer treatment. This commitment is not only reflected in the program’s day-to-day operations but is also evident in its collaboration with renowned institutions and experts in the field. In acknowledging the Livestrong Cancer Institute’s Survivorship Program, it becomes evident that its impact extends far beyond immediate healthcare concerns. It serves as a source of hope, empowerment, and resilience for cancer survivors worldwide, significantly contributing to the shift toward a more holistic and personalized approach in the ongoing evolution of cancer survivorship [26].

### 5.2. Cancer Survivorship Program at Memorial Sloan Kettering Cancer Center

Widely acknowledged for its exceptional standards in cancer care, the Cancer Survivorship Program at Memorial Sloan Kettering Cancer Center stands out as an example of comprehensive and personalized support. Going beyond the traditional boundaries of post-treatment care, the program is distinguished by its commitment to crafting individualized care plans, recognizing and addressing the unique journey each patient undergoes. At the core of this initiative is a holistic approach that transcends the medical aspects of survivorship, considering also the emotional, practical, and individualized needs of survivors. By tailoring ongoing support to the distinct requirements of each individual, Memorial Sloan Kettering’s Survivorship Program encapsulates an understanding of survivorship as a multidimensional experience. In addition to its emphasis on personalized care, the program offers a continuum of support that extends far beyond the immediate post-treatment phase. Memorial Sloan Kettering acknowledges the enduring challenges that survivors may face and, in response, provides a rich repository of educational resources. These resources not only inform patients about various aspects of their health but also empower them with the knowledge and tools needed to actively engage in their ongoing well-being. Moreover, the Program is deeply entrenched in the evolving paradigm of cancer survivorship. Its strong commitment to individualized care plans and ongoing support positions it at the forefront of shaping a patient-centric approach to post-treatment care. Collaborations with leading experts and institutions further highlight Memorial Sloan Kettering’s dedication to staying abreast of the latest advancements in survivorship care. In essence, this program emerges as a cornerstone in the landscape of survivorship care. Its unique blend of personalized care, continuous support, and educational empowerment highlights its pivotal role in enhancing the overall well-being of cancer survivors, making it an exemplary model in the dynamic terrain of cancer care [15].

### 5.3. Macmillan Cancer Support Survivorship Program

Macmillan Cancer Support Survivorship Program, based in the United Kingdom, emerges as a cornerstone in providing holistic support for cancer survivors. Recognized as a leading provider of practical, emotional, and financial assistance, Macmillan Cancer Support goes beyond immediate medical concerns to address the long-term impact of cancer on various aspects of life. At the heart of this program is a commitment to a comprehensive support system, acknowledging the multifaceted challenges that survivors may encounter post-treatment. Macmillan Cancer Support’s Survivorship Program stands as a reference for individuals navigating life after cancer, offering a range of services designed to enhance overall well-being. In addition to practical assistance, the program places a strong emphasis on emotional and financial support, recognizing that the aftermath of cancer extends beyond medical implications. By addressing the diverse and evolving needs of survivors, Macmillan Cancer Support contributes significantly to shaping a supportive and empathetic landscape for those on the journey beyond cancer. The Survivorship Program’s dedication to understanding and addressing the long-term impact of cancer makes it a vital resource for individuals seeking a comprehensive and compassionate support network. In the dynamic setting of cancer survivorship, Macmillan Cancer Support’s commitment stands as a testament to the evolution of post-treatment care [27].

### 5.4. Peter MacCallum Cancer Centre Survivorship Program

The Peter MacCallum Cancer Centre Survivorship Program, located in Australia, takes center stage as a dedicated provider of comprehensive support for cancer survivors. Positioned at the forefront of survivorship care, this program places a robust emphasis on the development of personalized survivorship care plans and the provision of ongoing support. With a commitment to tailoring services, the program strives to enhance both the physical and emotional well-being of cancer survivors. Beyond immediate medical concerns, the program recognizes the important and evolving needs of individuals post-treatment. By providing a personalized approach, it stands as a crucial resource for survivors navigating the multifaceted challenges of life beyond cancer. The emphasis on survivorship care plans and continual support aligns with the evolving paradigm of cancer care in Australia. The program’s dedication to enhancing both the physical and emotional aspects of survivorship reinforces its pivotal role in shaping a holistic and patient-centric approach to post-treatment care. In the dynamic landscape of cancer survivorship, the Peter MacCallum Cancer Centre Survivorship Program stands as an exemplar of the evolving narrative of support for those who have faced cancer [28].

### 5.5. Canadian Partnership against Cancer Survivorship Initiative

The Canadian Partnership Against Cancer Survivorship Initiative takes a prominent role in advancing survivorship care delivery across the nation. Led by the Canadian Partnership Against Cancer, this national initiative is dedicated to improving the landscape of survivorship care. In close collaboration with healthcare professionals, the program stands out for its focus on developing guidelines and resources that contribute to the provision of comprehensive survivorship care. Recognizing the importance of collaboration, the initiative engages healthcare professionals to ensure a collective and evidence-based approach to survivorship care. By developing guidelines and resources, the program not only aims to standardize care but also provides valuable tools for healthcare professionals to enhance the post-treatment experience for cancer patients. In the context of the evolving paradigm of cancer survivorship in Canada, the Initiative plays a pivotal role in shaping the trajectory of care. Its emphasis on collaboration, guideline development, and resource provision underlines its commitment to a comprehensive and patient-centric approach to survivorship care. In the dynamic landscape of cancer care in Canada, this initiative stands as a significant driver of positive change in the post-treatment journey of cancer survivors [29].

### 5.6. National Cancer Center Hospital Survivorship Program

In Japan, the National Cancer Center Hospital’s Survivorship Program takes a leading role in delivering comprehensive care with a special focus on psychological support. This program emerges as a dedicated initiative aimed at enhancing the quality of life for cancer survivors by addressing not only their medical but also their emotional needs. At the core of the program is a commitment to a holistic approach, recognizing that the post-treatment journey involves facing both the physical and psychological dimensions of survivorship. By providing comprehensive care that extends beyond medical concerns, the program exemplifies a dedication to fostering the overall well-being of cancer survivors. The inclusion of psychological support distinguishes this program, acknowledging the importance of addressing the emotional impact of survivorship. In the context of the evolving landscape of cancer care in Japan, the National Cancer Center Hospital’s Survivorship Program plays a crucial role in shaping a compassionate and patient-centered approach to post-treatment care. Its commitment to addressing both medical and emotional needs marks it as a vital resource for individuals navigating life after cancer [30].

### 5.7. Princess Margaret Cancer Centre Survivorship Program

The Princess Margaret Cancer Centre Survivorship Program, situated in Canada, distinguishes itself with a personalized approach to survivorship care plans. This program is dedicated to providing comprehensive support for cancer survivors by offering educational resources and ongoing assistance to empower individuals beyond their cancer journey. Central to the program is the development of personalized care plans, recognizing the uniqueness of each survivor’s experience. By tailoring support to the specific needs of individuals, the Princess Margaret Cancer Centre Survivorship Program is committed to empowering survivors with the knowledge and tools necessary for active participation in their ongoing well-being. In addition to personalized care, the program places a strong emphasis on education, providing resources that inform survivors about various aspects of their health. The commitment to ongoing support reflects an understanding of the enduring challenges that survivors may face post-treatment. In the dynamic landscape of cancer survivorship in Canada, this program contributes to shaping a personalized and empowering approach to post-treatment care. Its dedication to individualized care plans, educational resources, and ongoing support underscores its significance as a valuable resource for cancer survivors seeking comprehensive assistance in navigating life after cancer [31].

### 5.8. Cancer Council Australia’s Survivorship Program

Cancer Council Australia’s Survivorship Program appears as a comprehensive initiative, offering a wealth of resources and guidance tailored for cancer survivors. At the heart of this program is a commitment to ongoing support, providing valuable information to assist individuals experiencing the complexities of post-treatment life. The program is designed to be a steadfast companion for cancer survivors, recognizing that the post-treatment journey involves not only medical considerations but also multifaceted challenges. By emphasizing ongoing support, Cancer Council Australia’s Survivorship Program ensures that patients have access to a continuous source of assistance as they navigate the diverse aspects of life after cancer. In addition to its commitment to support, the program provides valuable resources that serve as a guide for survivors. This includes information on various facets of post-treatment life, empowering individuals with the knowledge needed to actively engage in their well-being. As a prominent player in the Australian landscape of cancer survivorship, Cancer Council Australia’s Survivorship Program significantly contributes to promoting a comprehensive and supportive approach to post-treatment care. Its emphasis on ongoing support and provision of valuable information highlights its role in enhancing the overall experience for individuals transitioning into life beyond cancer treatment [32].

In conclusion, the plethora of survivorship programs discussed exemplifies a global and strong commitment to enriching the post-treatment journey for cancer survivors. From the individualized care plans at Memorial Sloan Kettering Cancer Center to the comprehensive support provided by Livestrong, each program brings a unique perspective to survivorship care. The collective impact of these initiatives is evident in the standardization efforts by the American Cancer Society, the collaborative approach of the Canadian Partnership Against Cancer, and the community-building events across Europe. These programs not only address the physical and emotional aspects of survivorship but also contribute to the ongoing dialogue surrounding cancer care on a global scale. As we navigate the landscape of survivorship programs worldwide, it is clear that the evolution of comprehensive survivorship care continues to be shaped by the dedicated efforts of healthcare institutions, organizations, and advocates, all working toward enhancing the quality of life for those who have triumphed over cancer.

## 6. Strategies for Enhancing Cancer Survivorship Care: Addressing Gaps, Prevalent Comorbidities, and Innovations

Survivorship care for individuals overcoming cancer emerges as a critical aspect of post-treatment healthcare, at the same time revealing substantial gaps and limitations. Dr. Schapira has explicitly underscored these deficiencies, particularly emphasizing the need for integrating holistic care alongside conventional oncology services [33]. Building upon this perspective, Kemp et al. conducted a comprehensive study, as detailed in their research article titled “Mapping Systematic Reviews of Breast Cancer Survivorship Interventions: A Network Analysis” [34]. This study explores the landscape of breast cancer survivorship interventions, employing a network analysis approach. Kemp and colleagues highlight a significant imbalance within oncology services, with an overarching emphasis on prevention and recurrence surveillance. The study unveils that this emphasis often leads to the neglect of crucial aspects, such as psychological management, addressing comorbid conditions, and promoting overall health. Their findings underline the necessity of a more nuanced and inclusive approach to survivorship care. The research signals a prevailing industry focus on a survivor’s ability to endure, inadvertently sidelining the enduring impact on mental and physical health. Recognizing these gaps, as addressed by both Dr. Schapira and Kemp et al., is pivotal for shaping comprehensive strategies that prioritize the holistic well-being of cancer survivors. A concerted effort is needed to recalibrate this specific care, ensuring that it not only addresses the challenges of enduring the aftermath of the disease but also proactively fosters robust mental and physical health throughout the survivorship journey.

### 6.1. Prevalence and Impact

**Figure 2:**
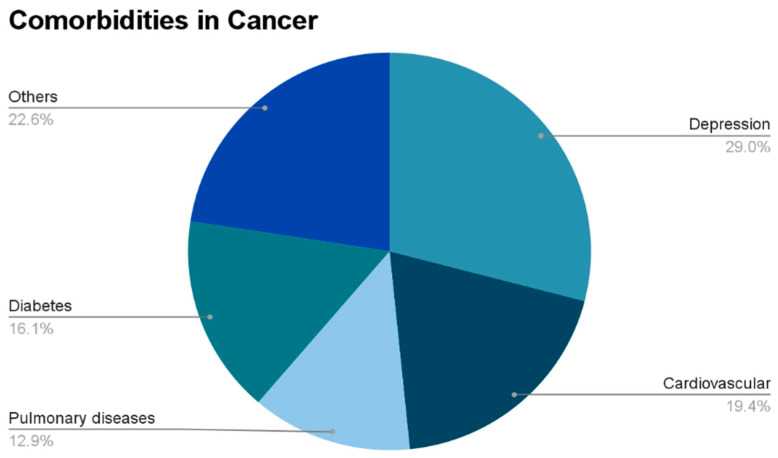
Pie chart showing various cancer comorbidities, with depression being the most common cause. Diabetes and cardiovascular and pulmonary diseases collectively account for over 45% of comorbidities. Data from a systematic review by Doose et al., 2022 [25].

The prevalence of comorbidities among cancer patients and survivors takes center stage (Figure 2), with Balic et al. [35] offering illuminating insights based on their study conducted in Austria. Their research, published in the Magazine of European Medical Oncology, revealed that a striking 86% of cancer patients slated for systemic anticancer treatment grapple with comorbid conditions. Among this cohort, 64% face the formidable challenge of managing at least two concurrent health issues. The study also underscored the nuanced impact of socioeconomic factors, such as deprivation, on the likelihood of multiple comorbidities, thereby emphasizing the importance of addressing broader systemic issues in cancer survivorship care. Complementing these findings, Panigrahi and Ambs [36] delve into the intricate relationship between comorbidities and cancer biology, presenting their insights in the Trends Cancer journal. Their research sheds light on how comorbidities not only contribute to the complexity of cancer biology but also play a pivotal role in shaping survival outcomes. Specifically, their work highlights the link between comorbid conditions and an increased risk of metastasis, emphasizing the need for a holistic understanding of cancer patient health. The study by Panigrahi and Ambs further highlights the imperative for survivorship care to extend beyond addressing the primary cancer diagnosis and actively manage and prevent concurrent health challenges. The intersection of socioeconomic factors with comorbidity prevalence, as evidenced by both studies, emphasizes the urgency of addressing health disparities in cancer survivorship care. Neglecting the comprehensive management of comorbid conditions not only jeopardizes the immediate well-being of the patient but also perpetuates disparities in long-term health outcomes. In light of these insights, it becomes evident that a robust and inclusive survivorship care strategy must comprehend not only the primary cancer-related concerns but also the proactive management of comorbid conditions, ensuring a more holistic approach to post-treatment healthcare.

### 6.2. Closing the Gaps

To effectively bridge the existing gaps in survivorship care, the implementation of innovative care delivery models is necessary. Dr. Schapira’s research underscores the significance of adopting risk-stratified survivorship care models, a paradigm that categorizes patients based on their health status to facilitate tailored care planning. This forward-thinking approach ensures the development of concise yet highly personalized care plans, precisely tailored to address the unique needs of individual survivors. Moreover, the integration of clinical group education sessions emerges as a resource-efficient strategy. These sessions not only serve as a platform for sharing valuable information but also foster a sense of community among survivors, encouraging shared experiences and mutual support. An exemplary embodiment of targeted care initiatives is evident in disease-specific clinics housed within comprehensive cancer centers, such as the breast cancer survivorship clinic at the Stanford Cancer Institute. These specialized clinics go beyond solely addressing medical needs; they create a supportive environment that recognizes and addresses the distinct challenges faced by survivors of specific cancer types. By combining medical expertise with empathetic support, these clinics offer a holistic approach to survivorship care, aiming to improve both the physical and emotional well-being of individuals post-cancer treatment. The integration of these innovative models signifies a significant shift toward a more comprehensive, patient-centered approach in the context of cancer survivorship care.

### 6.3. Shared Care Models

Explored in a systematic review by Doose et al. [25], shared care models integrate nurses, advanced practitioners, generalists, and specialists, showcasing consistently positive outcomes for cancer survivors with comorbidities. Published in the Journal of Healthcare Quality, the study provides insightful observations into the impact of team-based shared care on the quality of life and healthcare outcomes for this specific survivor population. The findings underline the efficacy of team-based shared care in addressing the intricate needs of cancer survivors, fostering a holistic approach through the collaboration of diverse healthcare professionals. Systematically reviewing existing literature, the study not only affirms positive outcomes but also contributes significantly to the evidence base supporting the adoption of shared care models in survivorship care. This integrated approach aligns seamlessly with the several aspects of survivorship care, recognizing the interplay between medical, emotional, and practical considerations. By cultivating a collaborative environment among professionals with varied specializations, shared care models represent a transformative shift toward a patient-centric paradigm. These insights, therefore, shed light on the significance of team-based care in providing a unified, effective, and compassionate healthcare approach for cancer survivors grappling with comorbidities.

### 6.4. Supportive Services

In the expansive landscape of cancer care, the significance of supportive services, particularly personalized symptom management, becomes even more evident when considering the comprehensive scientific statement authored by Gilchrist et al. [37] and published. This statement emphasizes the crucial role of cardio-oncology rehabilitation in managing cardiovascular outcomes for cancer patients and survivors. Collaborating across various disciplines, including the fields of exercise, cardiac rehabilitation, and secondary prevention, as well as clinical cardiology, cardiovascular and stroke nursing, and peripheral vascular disease, the research acknowledges the intricate relationship between cancer treatments and cardiovascular complications. Advocating for preventive strategies, the investigation aims to address late onset cardiological complications related to oncology treatments, ensuring the holistic well-being of cancer survivors. Furthermore, the paper underscores the importance of empowering survivors through knowledge dissemination and resource provision. This initiative goes beyond immediate symptom relief, promoting a proactive approach that enables survivors to actively participate in their ongoing care. The incorporation of cardio-oncology rehabilitation exemplifies a forward-thinking, multidisciplinary effort that aligns with a patient-centered focus within the broader spectrum of survivorship care. In essence, this scientific statement represents a milestone in recognizing and addressing the cardiovascular aspects of cancer survivorship. By advocating for proactive rehabilitation strategies, the initiative aims not only to enhance the immediate quality of life for cancer survivors but also to contribute significantly to the evolving landscape of comprehensive survivorship care.

In summary, the multifaceted challenges arising from comorbidities among cancer survivors necessitate a thoughtful and proactive approach. The integration of innovative, tailored, and shared care models emerges as a crucial strategy, promising a paradigm shift in survivorship care. By acknowledging the several and diverse needs of survivors and implementing precisely targeted interventions, healthcare providers have the opportunity to establish a continuum of care that transcends the treatment phase. This comprehensive and forward-thinking approach not only contributes to immediate improvements in outcomes and quality of life but also serves as a cornerstone for promoting the enduring well-being of cancer survivors throughout their entire journey and life.

### 6.5. Adolescent and Young Adult Survivors

Adolescents and young adult survivors (AYA) of childhood cancer are characterized by unique psychosocial, cognitive, and emotional challenges. They aim to achieve developmental milestones such as starting a family, having career aspirations, finding employment, and financial outcomes all compounded by their cancer experience. A cancer diagnosis may result in a struggle to find peers who can relate to their experiences, leading to a sense of alienation.

A study of 4804 patients that included children and AYAs aged between 2 and 39 years who had been newly diagnosed with cancer was conducted in North India from 1 April 2017 to 31 March 2019. A detailed clinical history, diagnosis, staging, treatment, outcomes, and follow-up were recorded for each patient [38]. According to the study, 60–70% of the study participants had high levels of stress that were significantly affecting the quality-of-life assessment (QOL). Furthermore, 65–75% of the patients had emotional problems, and the spiritual and religious problems were extremely prevalent, as well.

Fertility issues in AYA cancer survivors represent a significant concern, given the potential long-term impacts on their reproductive health and quality of life. According to a secondary analysis of a qualitative study of AYA survivors that was conducted, more female than male survivors were likely to feel distressed and overwhelmed; more female survivors also reported uncertainty about their fertility [39]. The major challenges that arose included fertility concerns, emotions raised when discussing fertility, and strategies used to manage fertility concerns. Fertility concerns focused on dating/partner reactions, health risks, and what potential infertility would mean for their life narrative, while emotions included distress, feeling overwhelmed, and wishful thinking.

Employment is considered a key aspect of cancer survivorship as it helps AYAs gain a sense of normalcy and helps them return to their normal lives. Unfortunately, AYA cancer survivors were employed significantly less often and had to apply for disability benefits. In addition, they were less likely to receive social assistance benefits—a benefit one can apply for in case of little or no income to pay for necessary costs [40]. AYA households were also significantly more dependent on receiving benefits as compared to achieving economic independence.

Support needs to be provided to AYAs in terms of fertility concerns, career aspirations, employment, and financial outcomes from diagnosis onwards as it may help AYAs find their way back into society.

## 7. Conclusions

Establishing a robust cancer survivorship program demands a holistic strategy, as delineated in the aforementioned strategies. To address the multifaceted needs of cancer survivors, the implementation of patient-centric programs therefore becomes necessary. This includes aspects such as educational initiatives, a strong support system, and avenues for connection. Furthermore, the provision of specialized clinical services, such as sexual health awareness, oncofertility, and psychological counseling, should always be present and personalized to embrace individual requirements. Overcoming the challenges associated with establishing networks with primary care providers and ensuring seamless care coordination is fundamental for fostering health promotion and integrating survivorship planning seamlessly with broader care plans [41].

In the matter of specific challenges, a study focused on breast cancer survivorship care revealed a concerning trend—only one-third of oncologists routinely screen survivors for subsequent primary cancers (SPCs), which constitute a significant cause of mortality among this population. These SPCs contribute to approximately 18% of new cancer cases in individuals with a cancer history. Despite the availability of disease-specific survivorship care guidelines, adherence to these recommendations in clinical practice often remains suboptimal. The gaps extend to understanding recurrence risks in diverse subgroups, including variations based on age, race, ethnicity, immigrant status, socioeconomic status, sexual identity, and rurality. Additionally, there is a need for improved recruitment of diverse participants for observational studies and clinical trials focused on surveillance [42,43]. Recognizing the significance of provider-initiated pain management communication, strategies involving pain assessments and prioritization of pain management in healthcare planning is crucial to enhancing survivor well-being [44].

To bridge the limitations in cancer care, ongoing research plays a crucial role in enhancing health outcomes. The primary target is centered around the imperative need to de-escalate cancer therapies to mitigate complications and toxicities. Precision oncology emerges as a key player in achieving this goal, with cancer genomics significantly advancing the field. The exploration of immunotherapies, currently under research, showcases phenomenal efficacy in treating specific cancer types. Transformative insights into tumor heterogeneity, cancer biology, and treatment resistance are arising through single-cell-level assays. Biomarker definition stands out as a burgeoning area of growth, contributing to the evolution of personalized cancer care [45,46]. Dr. Schapira strongly advocates for long-term tracking of studies exploring novel cancer therapies and understanding their late effects. A focus on comprehending the mechanisms of toxicity and optimizing the dosage of cancer therapy is essential. Additionally, interventions targeting behaviors and cognitive functions present promising avenues for improving outcomes in cancer survivors. A notable randomized controlled study further investigates and manages to demonstrate the efficiency of a mindset-focused digital intervention in enhancing the overall well-being of newly diagnosed nonmetastatic cancer patients [47].

### Enhancing Survivorship Care: Future Perspectives

Looking forward while acknowledging the steps achieved so far, comprehensive cancer survivorship care should consider perspectives from both patients and practitioners.

As we expect cancer survival rates to continue to show improvement over the next decade, the establishment of robust cancer survivorship care models becomes increasingly necessary. Among these models, the integration of patient-reported outcomes (PROs) emerges as a promising approach to facilitate supported self-care. PRO-driven assisted self-care enables patients to actively monitor their health status and symptoms using dedicated tools, while also promoting collaboration with healthcare practitioners for optimal care management [48]. This proactive approach not only enables earlier detection of health concerns but also facilitates tailored interventions, ultimately leading to improved outcomes. Moreover, by encouraging individuals to take an active role in managing their health, PRO-driven supported self-care promotes a sense of empowerment and control fundamental for patients.

The efficacy of such an approach is highlighted by several recent studies, such as one employing a web-mediated follow-up strategy utilizing self-reported symptoms. This approach demonstrated enhanced overall survival rates by enabling the prompt detection of relapses and improving performance status at relapse [49]. However, while revolutionary technological advancements hold great promise for enhancing cancer care, their integration must be considered with caution. Telemedicine, wearable health monitoring devices, and electronic patient education resources represent notable breakthroughs that can complement existing care practices. Nonetheless, challenges such as data privacy concerns, accessibility issues, and the risk of technology replacing human interaction in healthcare delivery warrant careful consideration [50].

In navigating these challenges, healthcare practitioners, particularly physicians and nurses, have a fundamental role in guiding the adoption of technological innovations in cancer care. While patient communities provide valuable feedback, healthcare providers bring essential clinical expertise to ensure that innovations are evidence-based and align with established best practices [51]. Finally, the successful incorporation of technological advancements into cancer care requires collaborative efforts among healthcare practitioners, patients, and technology developers. Such collaboration is essential to ensuring that innovations advance the quality of care without compromising the human-centered aspects of healthcare delivery.

In China, efforts in this direction are underway to train oncology specialists in providing tailored care based on the unique needs of cancer survivors.

This includes an emphasis on preventative screening modalities and rehabilitation efforts to improve not only cancer outcomes but also the quality of life for survivors. Far-sighted Dr. LS stresses the importance of early referrals, co-management across medical specialties, and empowering patients through social connections and timely access to healthcare. Raising awareness about cancer recurrence risk, increasing professional education through courses, and providing information to upcoming generations are essential for preparing primary healthcare for the growing population of cancer survivors [52].

As the landscape of cancer survivorship care continues to evolve, the integration and synergy of research findings, personalized interventions, and a collaborative approach will be pivotal. By addressing current gaps and challenges, exploring active research areas, and considering future perspectives, we can pave the way for a more comprehensive, effective, and patient-centric cancer survivorship care paradigm. The commitment to ongoing innovation, education, and collaboration ensures that the journey of cancer survivors is marked not only by survival but by thriving and achieving optimal well-being.
